# Assessment of the efficacy of two novel DNA vaccine formulations against highly pathogenic Porcine Reproductive and Respiratory Syndrome Virus

**DOI:** 10.1038/srep41886

**Published:** 2017-02-03

**Authors:** Luping Du, Fengjiao Pang, Zhengyu Yu, Xiangwei Xu, Baochao Fan, Kehe Huang, Kongwang He, Bin Li

**Affiliations:** 1 Institute of Veterinary Medicine, Jiangsu Academy of Agricultural Sciences, Key Laboratory of Veterinary Biological Engineering and Technology Ministry of Agriculture, Nanjing 210014, Jiangsu Province, China; 2 College of Veterinary Medicine, Nanjing Agricultural University, No. 1 Wei-gang, Nanjing 210095, China; 3 Jiangsu Co-infection Center for Prevention and Control of Important Animal Infectious Disease and Zoonoses, Yangzhou, Jiangsu Province 225009, China

## Abstract

Since May 2006, a highly pathogenic porcine reproductive and respiratory syndrome virus (HP-PRRSV) has emerged and prevailed in mainland China, affecting over 2 million pigs. Commercial PRRSV killed and modified live vaccines cannot provide complete protection against HP-PRRSV due to genetic variation. Development of more effective vaccines against the emerging HP-PRRSV is urgently required. In our previous studies, two formulations of DNA vaccines (pcDNA3.1-PoIFN-λ1-SynORF5 and BPEI/PLGA-SynORF5) based on the HP-PRRSV were constructed and shown to induce enhanced humoral and cellular immune responses in mice. The objective of this study was to evaluate the immune response induced by these novel formulations in piglets. PcDNA3.1-PoIFN-λ1-SynORF5 and BPEI/PLGA-SynORF5 vaccines induced significantly enhanced GP5-specific antibody and PRRSV-specific neutralizing antibody in pigs compared with the pcDNA3.1-SynORF5 parental construct. Though IFN-γ levels and lymphocyte proliferation responses induced by the two DNA vaccine formulations were comparable to that induced by the pcDNA3.1-SynORF5 construct, each of the novel formulations provided efficient protection against challenge with HP-PRRSV. Non-severe clinical signs and rectal temperatures were observed in pigs immunized with BPEI/PLGA-SynORF5 compared with other groups. Thus, these novel DNA constructs may represent promising candidate vaccines against emerging HP-PRRSV.

Porcine reproductive and respiratory syndrome (PRRS) is an economically frustrating viral disease of pigs, characterized by severe reproductive failure in pregnant sows, and respiratory disorders in piglets and growing pigs^1^. The disease was first detected in North America in 1987, then in Europe in 1990^2^. Since May 2006, a highly pathogenic (HP) PRRS has emerged in mainland China, characterized by high fever and high mortality in pigs of all ages, with a severe impact on the Chinese swine industry^3^,^4^,^5^. There are several available vaccines developed from HP-PRRS virus (HP-PRRSV) strains JXA1, HuN4, TJM and GDr; however, they have failed to provide efficient protection against the emerging HP-PRRSV strains^2^,^6^. Therefore, there is an urgent need to develop new vaccines against this emerging threat.

The causative agent, PRRSV, has a single-stranded, positive-sense RNA genome with a size of approximately 15 kb, which contains nine open reading frames (ORFs). GP5, the major envelope glycoprotein, plays important roles in virus infection, cell-binding and virus adsorption. GP5 is the key immunogenic protein, which can induce humoral and cellular immune responses^7^,^8^,^9^,^10^,^11^. In our previous research, we constructed a recombinant plasmid, pcDNA3.1-SynORF5, based on the HP-PRRSV JSKM strain. The DNA construct was then modified with the molecular adjuvant PoIFN-λ1 or BPEI/PLGA nanoparticles, which were demonstrated to improve the immunogenicity of the pcDNA3.1-SynORF5 DNA construct in mice^1^,^12^.

IFN-λ1 belongs to type III interferon family, which was firstly reported in 2003. It was reported that IFN-λ1 activated STATs (STAT1, STAT2, and STAT3), it contributed to the antiviral response by partly similar mechanisms as those used by IFN-α/β^13^, but when compared with type I interferon, its side effects are obviously little^1^. Poly(D, L-lactide-co-glycolide) (PLGA) nanoparticle-mediated delivery of vaccines has been shown to be effective in eliciting a protective immune response, and these particles can be administered by either the mucosal or the systemic route^14^,^15^. Polyethylenimine (PEI), as a cationic polymer, has been widely used to modify PLGA particles in order to enhance the efficiency of adsorption of DNA onto PLGA nanoparticles^12^.

Vaccines require optimal adjuvants, including immunopotentiator and delivery systems^16^,^17^, to offer long term protection from infectious diseases in animals. Immunopotentiators, such as cytokines, chemokines, chemical reagents and bacterial products, could enhance the immunogenicity of DNA vaccine formulations or subunit vaccines. Delivery systems (such as virus-like particles, liposomes, immune stimulating complexes, virosomes, microparticles and nanoparticles) present antigens to the immune system in an optimal manner by means which include the stabilization of antigen against degradation, controlled antigen release, targeting specific cells of the immune system (e.g. dendritic cells and macrophages), and co-delivery of antigen with immunopotentiators^18^,^19^,^20^.

In this study, we evaluated the immune responses in piglets induced by inoculation with one of two formulations of DNA vaccines, pcDNA3.1-PoIFN-λ1-SynORF5 and BPEI/PLGA-SynORF5, which were prepared during our previous research. The results obtained indicated that both formulations could efficiently protect against challenge with HP-PRRSV. According to our results, BPEI/PLGA-SynORF5 represents a potential candidate vaccine against HP-PRRSV.

## Results

### Humoral immune responses

**Figure 1:**
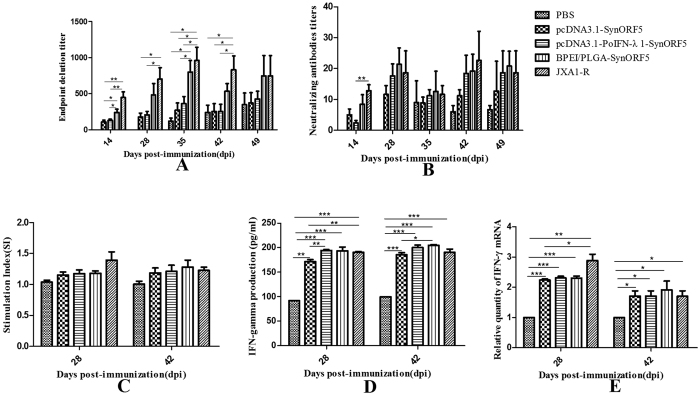
Detection of humoral and cellular immune response in pigs induced by PBS, pcDNA3.1-SynORF5, pcDNA3.1-PoIFN-λ1-SynORF5, BPEI/PLGA-SynORF5 or JXA1-R. The immunization protocol is described in the Methods. Serum samples were collected at 14, 28, 35, 42 and 49 dpi to determine the GP5-specific ELISA antibody (**A**) and neutralizing antibody against JSKM strain (**B**). The PBMCs were separated at 28 and 42 dpi and restimulated *in vitro* with purified PRRSV proteins (20 μg/mL). Lymphocyte proliferative assay (**C**), IFN-γ ELISA (**D**), and IFN-γ quantitative RT-PCR (**E**) were performed to assess the immune response. Data are presented as the mean ± S.E.M.

The immunogenicity of the two formulations of DNA vaccine was investigated in pigs. As shown in Fig. 1A, anti-PRRSV GP5-specific antibodies in pigs vaccinated with pcDNA3.1-SynORF5, pcDNA3.1-PoIFN-λ1-SynORF5, BPEI/PLGA-SynORF5 or commercial vaccine JXA1-R could be detected by ELISA at 14 dpi, and increased following a booster inoculation. At 14 and 28 dpi, no detectable GP5-specific antibodies were observed in pigs immunized with PBS. And at 35 dpi, BPEI/PLGA-SynORF5 induced significantly higher GP5-specific antibody titers compared with pcDNA3.1-SynORF5 (*P* = 0.0269) or PBS (*P* = 0.0479). At each sampling point, there was no significant difference in the level of GP5 antibody between the group immunized with the BPEI/PLGA-SynORF5 construct and JXA1-R. However, the titers of GP5 antibody in animals immunized with JXA1-R were significant higher than in those immunized with the pcDNA3.1-PoIFN-λ1-SynORF5 or pcDNA3.1-SynORF5 construct at 14, 28, 35 and 42 days post inoculation (dpi) (*P* < 0.01 or *P* < 0.05).

The neutralization capacity of sera from pigs was also investigated. As shown in Fig. 1B, PRRSV-specific neutralizing antibodies in pigs vaccinated with pcDNA3.1-SynORF5, pcDNA3.1-PoIFN-λ1-SynORF5, BPEI/PLGA-SynORF5 or commercial vaccine JXA1-R were detected on the 14^th^ day after primary immunization and elevated by the 28^th^ day. Meanwhile, no neutralizing antibodies (<1:2) against HP-PRRSV strain JSKM were detected in the PBS control group on the 14^th^ or 28^th^ days. Only at 14 dpi, was there a significant difference in the titer of neutralizing antibodies between the groups immunized with pcDNA3.1-PoIFN-λ1-SynORF5 and JXA1-R (*P* = 0.0011).

### Lymphocyte proliferation and cytokine detection in pigs

At 28 and 42 dpi, peripheral blood mononuclear cells (PBMCs) were isolated and re-stimulated *in vitro* with purified PRRSV (20 μg/mL) to analyze cellular immune responses. As shown in Fig. 1C, at 28 and 42 dpi, there was no significant difference in lymphocyte proliferative responses among the groups immunized with pcDNA3.1-PoIFN-λ1-SynORF5 or BPEI/PLGA-SynORF5 constructs, or JXA1-R (*P* > 0.05). At 28 and 42 dpi, the stimulation index (SI) detected in the groups immunized with pcDNA3.1-PoIFN-λ1-SynORF5 or BPEI/PLGA-SynORF5 constructs, or JXA1-R were numerically higher than the group vaccinated with PBS, but the differences were not statistically significant (*P* > 0.05).

To profile the cytokine levels induced by the DNA plasmids, IFN-γ secretion in splenocytes restimulated with PRRSV protein was measured by ELISA. As depicted in Fig. 1D, at 28 and 42 dpi, there were no significant difference in the level of IFN-γ production among the groups immunized with pcDNA3.1-PoIFN-λ1-SynORF5 or BPEI/PLGA-SynORF5 constructs, and JXA1-R (*P* > 0.05). And at 42 dpi, the IFN-γ production induced by BPEI/PLGA-SynORF5 was significantly higher than that induced by pcDNA3.1-SynORF5 (*P* = 0.0105). At 28 and 42 dpi, the production of IFN-γ in the control group was the lowest one when compared with other vaccinated groups, and the differences were significant (*P* < 0.01 or *P* < 0.001). Quantitative real-time RT-PCR was also performed to analyze the level of Th1 cytokine IFN-γ mRNA expression in the restimulated splenocytes. Consistent with the results of the IFN-γ ELISA assay, at 28 and 42 dpi, there were no significant difference in the level of IFN-γ mRNA expression among the groups immunized with pcDNA3.1-PoIFN-λ1-SynORF5 or BPEI/PLGA-SynORF5 constructs, and JXA1-R (*P* > 0.05). And significant differences could be observed between the control group and other vaccinated groups (*P* < 0.05, *P* < 0.01 or *P* < 0.001) (Fig. 1E).

### CD4^+^ and CD8^+^ T-cell subtype assays

**Figure 2:**
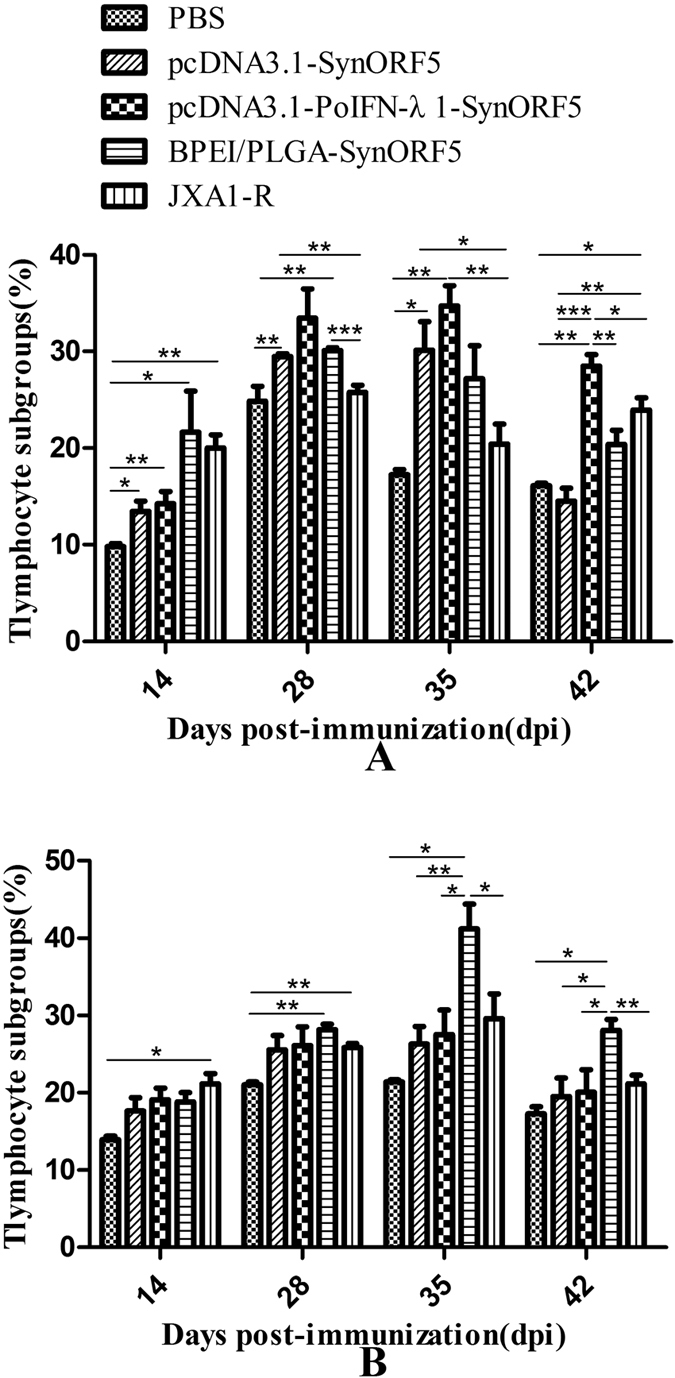
Flow cytometric analysis of proportions of CD3^+^ CD4^+^ (**A**) and CD3^+^ CD8^+^ T-lymphocyte subpopulations in fresh PBMCs isolated from different groups of vaccinated pigs at 14, 28, 35, and 42 dpi. Results were obtained by averaging data from the five serum samples of each group (n = 5). Data are shown as mean ± S.E.M.

Lymphocytes were isolated at 14, 28, 35 and 42 dpi, and analyzed for CD3^+^ CD4^+^ and CD3^+^ CD8^+^ T lymphocytes by flow cytometry. As shown in Fig. 2, all prepared formulations of DNA vaccine induced higher levels of CD3^+^ CD4^+^ and CD3^+^ CD8^+^ T cells after primary immunization. The percentage of CD3^+^ CD4^+^ T cells in the BPEI/PLGA-SynORF5 group was significantly higher compared with the JXA1-R-vaccinated group at 28 dpi (*P* = 0.0007). And significant differences in the level of CD3^+^ CD8^+^ T cells could be observed between BPEI/PLGA-SynORF5 and JXA1-R groups at 35 and 42 dpi (*P* = 0.0331 and *P* = 0.0047). At 28 dpi, there were significant differences in the percentage of CD3^+^ CD4^+^ and CD3^+^ CD8^+^ T cells between the groups immunized with PBS and BPEI/PLGA-SynORF5 (*P* = 0.003 and *P* = 0.0019). After challenge, the number of CD3^+^ CD4^+^ T cells decreased at 35 dpi before increasing slightly at 42 dpi in the pcDNA3.1-PoIFN-λ1-SynORF5 and BPEI/PLGA-SynORF5 groups, which was in opposition to the results observed in the JXA1-R group. By contrast, the number of CD3^+^ CD8^+^ T lymphocytes in the pcDNA3.1-PoIFN-λ1-SynORF5, BPEI/PLGA-SynORF5 and JXA1-R groups increased at 35 dpi. But at 42 dpi, it decreased to a similar level to that detected at 14 dpi.

### Protective efficiency against PRRSV challenge

#### Clinical signs and body temperature change

**Figure 3:**
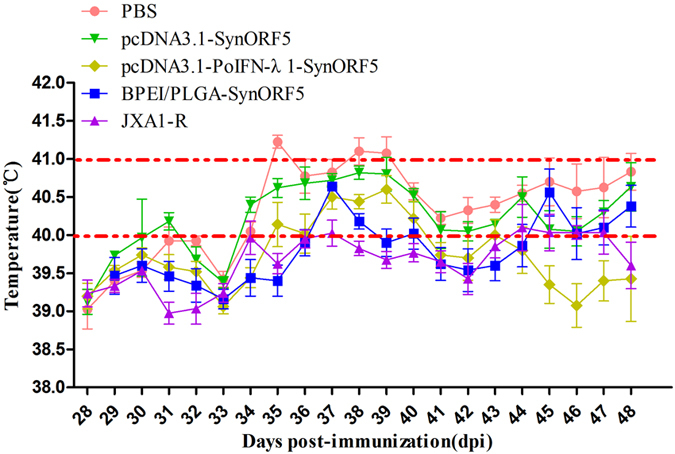
Rectal temperature of pigs in each group. Rectal temperature of each pig was measured daily after PRRSV challenge. Data are shown as mean ± S.E.M.

After challenge with virulent PRRSV, all pigs in the PBS control group had high rectal temperature (≥40 °C) (Fig. 3). At 35, 38 and 39 dpi, the temperature of the control group was as high as 41 °C and pigs displayed a range of clinical signs, including inappetence, lethargy, rough hair coats, dyspnea, periocular edema, eyelid edema and light diarrhea. Though pigs in the pcDNA3.1-PoIFN-λ1-SynORF5 group had high rectal temperature during 37–40 dpi, the mean temperature at each point was lower than the group immunized with pcDNA3.1-SynORF5, and presented with only slight depression in terms of clinical signs. Pigs in the BPEI/PLGA-SynORF5 group had high rectal temperature (≥40 °C) only at 37 and 38 dpi, and no obvious clinical signs were observed in this group. Throughout the observation period after challenge, no pigs in the JXA1-R group had high rectal temperature (≥40 °C), and no obvious clinical signs were observed.

Furthermore, scores for clinical signs in the BPEI/PLGA-SynORF5 group were significantly lower than those in the PBS and pcDNA3.1-SynORF5 groups (*P* = 0.0045 and *P* = 0.0104) (Table 1).

#### Viremia and tissue viral loads after challenge

**Figure 4:**
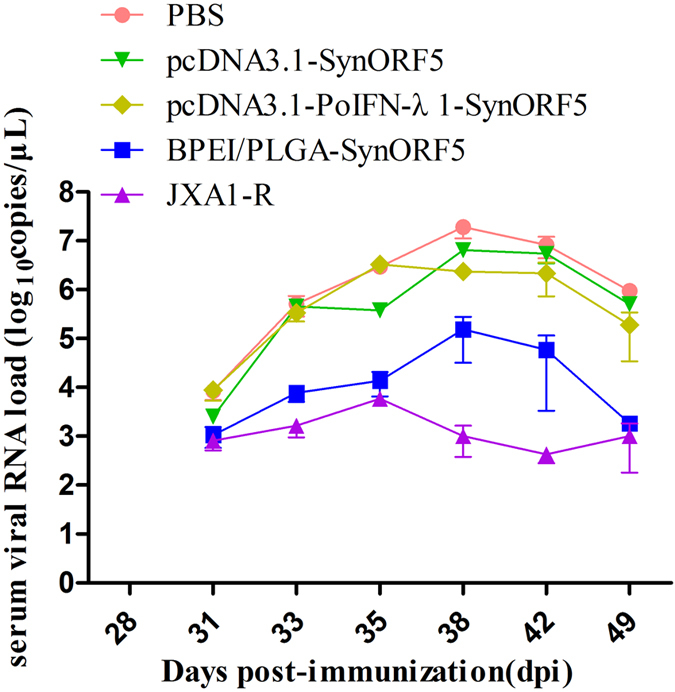
Viremia of pigs inoculated with different vaccine formulations followed by challenge with the PRRSV JSKM strain. Viral RNA of serum from each pig in different groups (n = 5) was detected.

**Figure 5:**
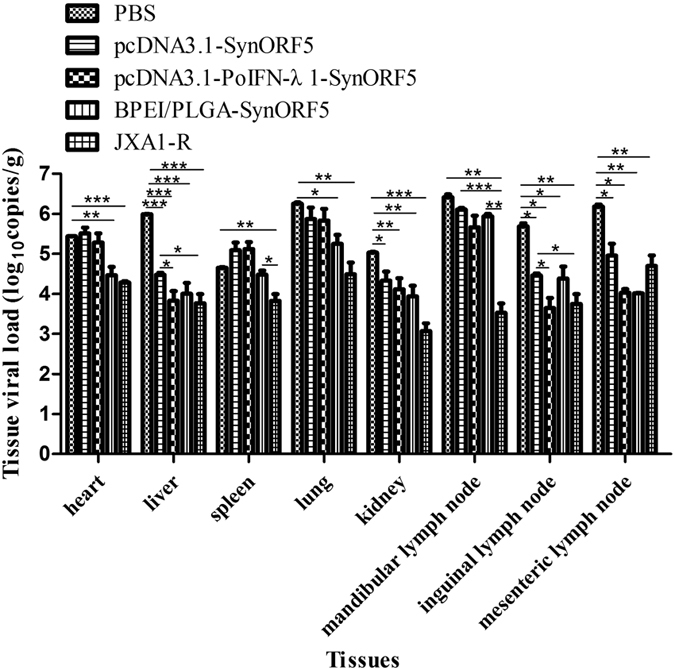
Tissue viral loads of pigs inoculated with different vaccine formulations followed by challenge with the PRRSV JSKM strain. Viral RNA in serum from each pig in different groups (n = 5) was detected.

During the 21 days after challenge with JSKM virus, pigs inoculated with BPEI/PLGA-SynORF5 showed a lower level of viremia in blood than that of the control PBS group (*P* < 0.05), and no significant difference when compared with pigs in the JXA1-R-vaccinated group (*P* > 0.05) (Fig. 4). However, a similar level of viremia in blood was observed between the groups immunized with pcDNA3.1-PoIFN-λ1-SynORF5 and pcDNA3.1-SynORF5. Even the similar level of viremia in blood was observed among the groups vaccinated with pcDNA3.1-PoIFN-λ1-SynORF5, pcDNA3.1-SynORF5 and PBS at 31 and 33 dpi, at other sampling times, the level of viremia in blood of pigs inoculated pcDNA3.1-PoIFN-λ1-SynORF5 and pcDNA3.1-SynORF5 showed numerically lower than that receiving PBS. At the end of the study, all pigs were necropsied, and viral loads in different tissues were determined. Tissues collected from pigs inoculated with pcDNA3.1-SynORF5 and pcDNA3.1-PoIFN-λ1-SynORF5 exhibited much higher viral loads in the heart, spleen, lung, and mandibular lymph node (Fig. 5). However, in the liver, inguinal lymph node and mesenteric lymph node, pigs vaccinated with pcDNA3.1-PoIFN-λ1-SynORF5 showed similar or lower levels in viral loads compared with the group inoculated with JXA1-R.

Although virus could still be detected in the tissues of all pigs at the end of the experiment, the viral loads in the heart, liver, lung, kidney, inguinal lymph node and mesenteric lymph node from pigs of the BPEI/PLGA-SynORF5 group were significantly lower than those from PBS group. And there was no significant difference in viral loads of the sampling tissues between the groups immunized with BPEI/PLGA-SynORF5 and JXA1-R.

#### Pathological examination

**Figure 6:**
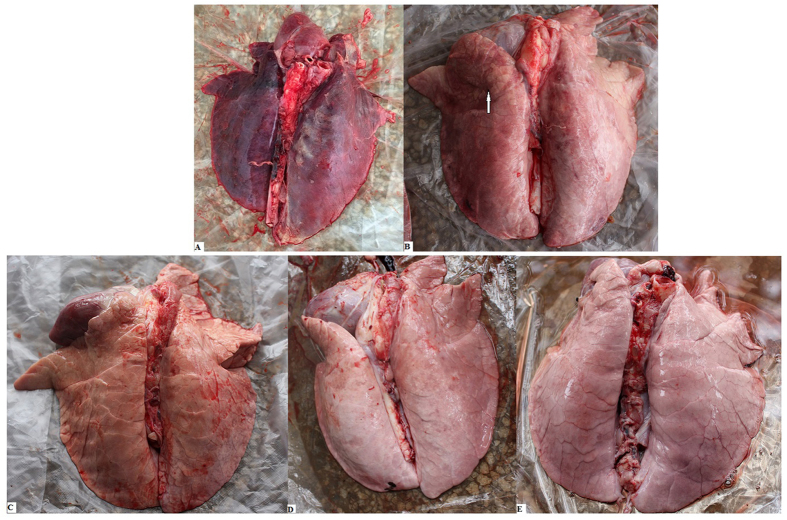
Macroscopic lesions in lungs of pigs in the PBS (**A**), pcDNA3.1-SynORF5 (**B**), pcDNA3.1-PoIFN-λ1-SynORF5 (**C**), BPEI/PLGA-SynORF5 (**D**) and JXA1-R (**E**) groups at 49 dpi. Macroscopic lesions in the PBS group piglets were more severe including interstitial pneumonia with consolidation and hemorrhage. Interstitial pneumonia with consolidation (hollow arrow) was observed in the pcDNA3.1-SynORF5 group. No obvious pathological changes were observed in the lungs of pcDNA3.1-PoIFN-λ1-SynORF5, BPEI/PLGA-SynORF5 and JXA1-R-vaccinated piglets.

**Figure 7:**
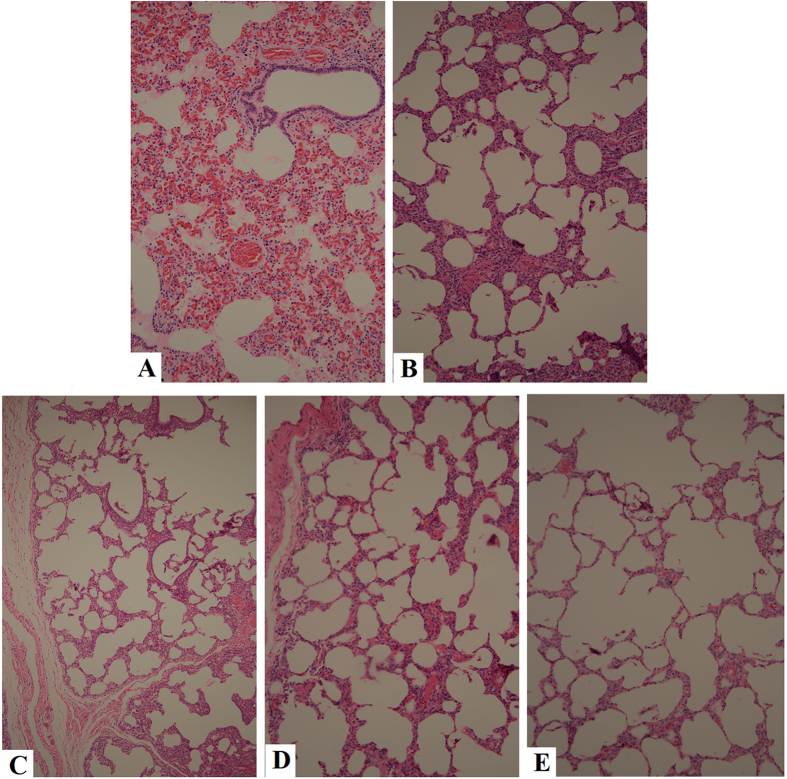
Examination of histological lesions in lungs of pigs in the PBS (**A**), pcDNA3.1-SynORF5 (**B**), pcDNA3.1-PoIFN-λ1-SynORF5 (**C**), BPEI/PLGA-SynORF5 (**D**) and JXA1-R (**E**) groups at 49 dpi. Analysis indicated that interstitial pneumonia of pigs in both pcDNA3.1-PoIFN-λ1-SynORF5, BPEI/PLGA-SynORF5 and JXA1-R groups was much milder than that in the PBS and pcDNA3.1-SynORF5 groups. Magnification 200×.

At 49 dpi, the lungs were examined for gross lesions in all lobes. As shown in Fig. 6, macroscopic lesions were mainly found to occur in the lungs of pigs receiving PBS. The gross pathological changes were characterized by interstitial pneumonia with consolidation and hemorrhage. Interstitial pneumonia with consolidation was observed in the pcDNA3.1-SynORF5 group. However, no obvious pathological changes in the lungs of pcDNA3.1-PoIFN-λ1-SynORF5, BPEI/PLGA-SynORF5 or JXA1-R-vaccinated piglets were observed. On histological examination, lung lesions in control pigs were characterized by thickened alveolar walls, extensive infiltration of macrophages, and increased amounts of bronchiole exudate. Interstitial pneumonitis could be observed in the pcDNA3.1-SynORF5 and pcDNA3.1-PoIFN-λ1-SynORF5 groups, which was much milder than that in the PBS groups. However, this interstitial pneumonitis was largely absent in the BPEI/PLGA-SynORF5 and JXA1-R-vaccinated animals (Fig. 7).

## Discussion

Since May 2006, a highly pathogenic strain of PRRSV with a unique 30-amino-acid deletion in its Nsp2 coding region has emerged and prevailed in mainland China, leading to excessive economic losses in the swine industry of China^3^. Even though vaccines against HP-PRRSV have been developed and marketed to the Chinese swine industry, the disease has remained prevalent because of the antigenic variations in HP-PRRSVs. Thus, it is imperative to develop vaccines against the emerging HP-PRRSV strains in order to provide protective immune responses in pigs.

At present, Commercial PRRSV vaccines including killed and modified live ones are available. However, both of them have inherent drawbacks. Killed vaccines are weakly immunogenic and MLVs has the potential revert to high virulence^21^,^22^,^23^. DNA vaccines are the third revolution in the vaccine field, and have been found to be safe to the host while generating both humoral and cellular immunity^24^,^25^. Moreover, DNA vaccines can be easily modified to enhance the poor immunogenicity. Adjuvants including immunopotentiator and delivery systems were necessary for the development of DNA vaccines. In order to increase the efficiency of the PRRSV vaccines, they were modified with immunopotentiators, including HSP70^26^, IL-18^8^, GM-CSF^27^, CD40L^28^, CpG oligodeoxynucleotides^29^,^30^, IL-2^31^, CTLA4^32^ and interferon α/β^33^. Furthermore, delivery systems were also applied to the design of PRRSV vaccines, for example, viral vectors^34^,^35^,^36^, chitosan^25^,^37^ and poly(lactic-co-glycolic acid) (PLGA)^38^,^39^,^40^,^41^. In our previous studies, an immunopotentiator (PoIFN-λ1) and delivery system (BPEI/PLGA) were applied to modify pcDNA3.1-SynORF5 constructs, based on the HP-PRRSV JSKM strain. We demonstrated that both formulations of DNA vaccine could improve the immunogenicity of pcDNA3.1-SynORF5 in a mouse model. Thus, in this study, we chose to evaluate the immune response induced following inoculation of piglets with the two DNA vaccine formulations to establish an attractive candidate vaccine against HP-PRRSV. To our knowledge this is the first report of a comparative efficacy test of two formulations of adjuvants (immunopotentiator and delivery system) used to modify a PRRSV DNA vaccine in pigs.

Neutralizing antibody was widely thought as a valuable parameter to evaluate the efficacy of a vaccine against PRRSV infection^42^,^43^,^44^. Likewise, cellular immune response, particularly the level of virus-specific IFN-γ, has been another potential correlate of protective immunity against PRRSV^45^,^46^.

In this study, as shown in Fig. 1, higher titers of GP5 antibody and neutralizing antibody were induced by BPEI/PLGA-SynORF5 vaccine when compared with parental construct pcDNA3.1-SynORF5. The similar IFN-γ levels or lymphocyte proliferative responses could be observed among the groups immunized with pcDNA3.1-PoIFN-λ1-SynORF5, BPEI/PLGA-SynORF5 or JXA1-R, which implies that similar levels of cellular immunity can be induced by the prepared vaccine candidates and the commercial vaccine JXA1-R, it was higher than the PBS group. The pcDNA3.1-PoIFN-λ1-SynORF5 and BPEI/PLGA-SynORF5 constructs did, however, drive stronger T cell differentiation compared with the pcDNA3.1-SynORF5 construct as early as 14 days after primary immunization. These results are consistent with previous studies by Dwivedi *et al*. and Binjawadagi *et al*.^38^,^39^,^40^,^41^.

To investigate the level of protection elicited by pcDNA3.1-PoIFN-λ1-SynORF5 or BPEI/PLGA-SynORF5, all the test pigs were challenged at 28 dpi with the HP-PRRSV JSKM strain. The results presented showed that all pigs in the control group showed more severe clinical signs than the vaccinated groups. Rectal temperatures of all test groups were coincident with the viremia detected by real-time RT-PCR. No obvious clinical signs or pathological changes could be observed in the groups inoculated with BPEI/PLGA-SynORF5 or JXA1-R. Given these observations, BPEI/PLGA-SynORF5 may provide more effective protection for pigs, although the viremia could not be fully prevented after virus challenge. Although virus could still be detected in the tissues of all pigs at the end of the experiment, viral loads in the heart, liver, lung, kidney, inguinal lymph node and mesenteric lymph node from pigs in the BPEI/PLGA-SynORF5 group were significantly lower than those from the PBS group, which indicates that piglets inoculated with BPEI/PLGA-SynORF5 can successfully restrict viral replication in tissues. Moreover, a ten-fold reduction in viral RNA load could be observed in the lungs of animals immunized with BPEI/PLGA-SynORF5 compared with the PBS control group.

Results from our study showed that JXA1-R was successful in reducing the clinical disease such as temperatures, lung lesions, viral load, but JXA1-R, belonging to MLVs was invariably implicated in spreading the mutated viruses to susceptible pigs^22^. Moreover, there are reports of reversion of vaccine virus into virulence leading to severe disease outbreaks, and like the field virus, PRRS-MLV also induces immunosuppression. However, DNA vaccine, without reversion to high virulence, was safe to the host while generating both humoral and cellular immunity and could be easily modified with immunopotentiator and delivery systems. The constructs of PRRSV with the potential to induce immunosuppression could be avoided when designing PRRSV DNA vaccine. Based on our current results, we can also modify pcDNA3.1-SynORF5 by inserting PRRSV ORF3 and ORF6, with the potential to induce protective immune responses.

The results of this study demonstrate that the DNA vaccine, BPEI/PLGA-SynORF5, could elicit immune responses against a homologous PRRSV challenge, although protection against heterologous virus challenge should be further investigated for the rational design of future vaccine strategies. Studies on adjuvanted PLGA nanoparticle-entrapped inactivated PRRSV vaccine have demonstrated that it could provide cross-protective immunity against PRRSV in pigs^38^,^39^,^40^,^41^. As we can modify pcDNA3.1-SynORF5 by inserting other genes encoding immunogenic proteins or heterologous PRRSV ORF5 genes, we speculated that PRRSV DNA vaccine adjuvanted with PLGA nanoparticles may be a potential vaccine candidate, and thus demonstrated that BPEI/PLGA nanoparticles show potential for use in development of PRRSV vaccines.

In our study, the pcDNA3.1-SynORF5 plasmid was adsorbed on the BPEI/PLGA nanoparticles, whereas in the work completed by Dwivedi and Binjawadagi, the inactivated PRRSV vaccines were entrapped in PLGA nanoparticles, which may affect the physical and chemical stability of antigens during the progress of nanoparticle preparation. The method of adsorption would protect antigens from this limitation. Furthermore, BPEI was utilized to modify the PLGA nanoparticle in order to improve the efficiency of adsorption. Compared with other DNA vaccine of PRRSV such as pCA-U-optiGP5^9^, pcDNA-5L6, pcDNA-6L5^47^, and pVAX1-EU-ORF3-ORF5^37^, BPEI modified PLGA nanoparticles could improve the delivery of adsorbed DNA into antigen-presenting cells after i.m. injection. Although these DNA vaccines could enhance the immune response, they didn’t provide enough protection after challenge. It is believed that adsorption of the DNA vaccine on BPEI/PLGA allows it to be presented in particulate form, which appears to be more antigenic and can be efficiently phagocytosed^48^.

In summary, we evaluated the immune response induced by two formulations of PRRSV DNA vaccine (pcDNA3.1-PoIFN-λ1-SynORF5 and BPEI/PLGA-SynORF5) following inoculation of piglets, and the protection after challenge with an HP-PRRSV JSKM variant. Both the pcDNA3.1-PoIFN-λ1-SynORF5 and BPEI/PLGA-SynORF5 formulations induced enhanced immune responses compared with the parent construct pcDNA3.1-SynORF5, and provided protection against challenge with HP-PRRSV JSKM strains. BPEI/PLGA-SynORF5 elicited the strongest responses and thus may provide the most efficient protection against PRRSV infection among the parent construct pcDNA3.1-SynORF5, pcDNA3.1-PoIFN-λ1-SynORF5 and BPEI/PLGA-SynORF5. And BPEI/PLGA-SynORF5 may represent promising candidate vaccines against PRRSV.

## Methods

### Plasmids, cells and virus

Plasmid pcDNA3.1-SynORF5, based on the native ORF5 gene of HP-PRRSV strain JSKM (GenBank accession number HQ832104), pcDNA3.1-PoIFN-λ1-SynORF5 and Marc-145 cells were maintained in our laboratory. HP-PRRSV strain JSKM, isolated from the lungs of a pig infected with the “high fever” syndrome in Jiangsu Province, was propagated and titrated in Marc-145 cells, as previously described^49^. Large-scale preparations of plasmid DNA, including pcDNA3.1, pcDNA3.1-SynORF5, and pcDNA3.1-PoIFN-λ1-SynORF5, were purified by Endofree Maxi Plasmid Kit (TIANGEN, Beijing, China), as per the manufacturer’s instructions. The plasmids were adjusted to a final concentration of 1 μg/μL. BPEI/PLGA-SynORF5 was prepared by a modified double emulsion solvent evaporation technique according to Du *et al*.^12^.

### Animal study design

Twenty-five piglets weaned at 3 weeks of age were obtained from a PRRS-free farm in Nanjing. The piglets were confirmed negative for PRRSV by RT-PCR and ELISA (IDEXX). The piglets were then randomly separated into five groups, housed in separate rooms at the animal facility of the Institute of Veterinary Medicine, Jiangsu Academy of Agricultural Sciences, Nanjing, Jiangsu Province. Three groups were vaccinated intramuscularly twice at 2-week intervals with pcDNA3.1-PoIFN-λ1-SynORF5, pcDNA3.1-SynORF5 or BPEI/PLGA-SynORF5 dissolved in 1 mL of PBS, each containg 500 μg plasmids. The fourth group was injected intramuscularly once with commercial PRRSV attenuated vaccine (HP-PRRS vaccine, live strain JXA1-R, Dahuanong, Guangdong, China). The control group was injected intramuscularly with 1 mL of PBS. Serum samples were collected from each pig at 14, 28, 35, 42 and 49 dpi to detect specific anti-PRRSV antibodies. At 28 dpi, fresh PBMCs were collected from inoculated pigs for T lymphocyte proliferation, IFN-γ release and T cell subset (CD3^+^ CD4^+^ and CD3^+^ CD8^+^) assays.

All groups were challenged intramuscularly at 28 dpi with PRRSV JSKM (2 mL) with an infectious titer of 1 × 10^5^ 50% tissue culture infectious dose per mL. Animals were monitored for an additional 21 days, and rectal temperatures and clinical signs were observed daily. Blood was collected at 28, 31, 33, 35, 38, 42 and 49 dpi for viremia and antibody detection. At 42 dpi, fresh lymphocytes were separated from the peripheral blood of piglets to detect specific cell-mediated immune responses. At 49 dpi, all pigs were euthanized for pathological analysis.

### Serological tests

GP5-specific antibodies were determined with an endpoint ELISA using purified recombinant GP5 as the antigen, as described previously^50^. Titers were expressed as the reciprocal of the highest dilution of sera producing ratio values of 2.1. Serum neutralization assays were performed as described by Ostrowski *et al*.^51^. The neutralization titers were expressed as the reciprocal of the highest serum dilution resulting in complete neutralization. Each sample was run in triplicate.

### Lymphocytes proliferation assay

Lymphocyte proliferation assays were performed using PBMCs from the experimental animals. Peripheral blood mononuclear cells were collected and stimulated with or without 20 μg/mL PRRSV proteins, which were prepared by ultracentrifugation of Marc-145 cells infected with PRRSV strain JSKM at 80,000 × g for 2 h. Lymphocyte proliferation assays were performed as described previously^52^. The SI was calculated as the ratio of the average OD value of wells containing antigen-stimulated cells to the average OD value of wells containing cells with medium only.

### IFN-γ release assay

The isolated lymphocytes (1 × 10^6 ^cells/mL) were cultured in 24-well plates at 37 °C with 5% CO_2_, with or without 20 μg/mL PRRSV proteins. After 72 h incubation, culture supernatant was harvested and the presence of IFN-γ was tested with a commercial porcine IFN-γ immunoassay ELISA kit according to the manufacturer’s instructions (USCN Business Co., Ltd., Wuhan, China). The concentrations of IFN-γ in the samples were determined from standard curves.

### Real-time PCR analysis of IFN-γ mRNA expression

Peripheral blood mononuclear cells (1 × 10^6 ^cells/mL) were cultured in 24-well plates for 18 h at 37 °C in the presence of 5% CO_2_, with or without 20 μg/mL PRRSV proteins. Total RNA was extracted by Trizol (Intrivogen, USA) and quantified by Ultraviolet spectrophotometer. Then 1 μg of RNA was reverse transcribed in a 20 μL reaction mixture, with 4 μL 5 × QRT Super Mix (Vazyme, Nanjing, China)^2^. The cDNA product (0.5 μL) was amplified in a 25 μL reaction mixture containing SYBR® *Premix Ex Taq*™ (Tli RNaseH Plus) (2 × ) (12.5 μL) (Takara, Dalian, China), ROX Reference Dye II (50 × ) (0.5 μL) (Takara, Dalian, China), and 0.2 μM each of the forward and reverse gene-specific primers (Pig IFN-γ: AGAATTGGAAAGAGGAGAGTGACAA/TGAATGGCCTGGTTATCTTTGA; Pig-GAPDH: ACATGGCCTCCAAGGAGTAAGA/GATCGAGTTGGGGCTGTGACT). Each reaction was performed in triplicate. Real-time PCR amplifications were performed using an Applied Biosystems 7500 Real-Tine PCR System. Thermal cycling conditions were 2 min at 50 °C, 10 min at 94 °C, followed by 40 cycles of 15 s at 94 °C and 1 min at 60 °C. Gene expression was measured by relative quantity as described previously^36^.

### Analysis of CD4^+^ and CD8^+^ T-lymphocytes

At 14, 28, 35 and 42 dpi, peripheral blood was collected from each pig. One million PBMCs were separated and transferred into a 1.5 mL centrifuge tube. One milliliter of a fluorescence solution (100 mL 0.15 M PBS pH 7.4, 2% newborn bovine serum) was then added, and the tube was centrifuged at 170 × g for 3–5 min. The supernatant was removed, and the pellet was resuspended in 300 μL of cell fluorescence solution for staining with FITC mouse anti-porcine CD3, PE mouse anti-porcine CD8 and SPRD mouse anti-porcine CD4 (SouthernBiotech, Birmingham, AL) fluorescent antibodies at 4 °C in the dark for 30 min. After washing twice with fluorescence solution and centrifugation at 170 × g for 5 min, the supernatant was discarded. The cell pellet was resuspended in 500 μL of fluorescence preservation solution (0.15 M PBS pH 7.4, 2% glucose, 1% formaldehyde, 0.1% NaN_3_). Flow cytometry was then used to count CD3^+^ CD4^+^ and CD3^+^ CD8^+^ T-lymphocytes in 10,000 cells, and percentages of CD3^+^ CD4^+^ and CD3^+^ CD8^+^ T-lymphocytes were determined.

### Clinical evaluations

The severity of clinical signs was evaluated daily after challenge as reported previously^53^. Briefly, the clinical condition of the experimental animals was evaluated based on a numerical index that reflected the severity of illness. Scores for each of three individual observations ranged from 1 to 4. The observation score consisted of the sum of the daily observations for behavior, respiration and cough. For example, a clinically normal animal would be given a total score of 3 (i.e., behavior = 1, respiration = 1, cough = 1), an animal with maximum clinical illness would be given a total score of 9 (i.e., behavior = 3, respiration = 3, cough = 3) and a dead animal would be given a total score of 12 (i.e., behavior = 4, respiration = 4, cough = 4).

### Measurements of serum and tissue virus loads in piglets

At 49 dpi, all pigs were euthanized, blood was drained and tissue samples were collected for pathological examination. To further quantitatively evaluate viral replication *in vivo*, real-time RT-PCR based on the ORF7 sequence was performed to determine the PRRSV load in the serum of inoculated pigs at 28, 31, 33, 35, 38, 42 and 49 dpi, and in tissues collected at necropsy (heart, liver, spleen, lung, kidney, mandibular lymph node, inguinal lymph node and mesenteric lymph node).

Total RNA in the serum samples and tissues were extracted by Trizol. Real-time PCR amplification to detect viremia was carried out with Probe qRT-PCR supermix (Takara, Dalian, China) in a 20 μL reaction mixture containing 10 μL of 2 × Supermix, 10 μM of primers (F: 5′-TCAGCTGTGCCAAATGCTGG-3′; R: 5′-AAATGGGGCTTCTCCGGGTTTT-3′), 10 μM of probe (5′-FAM- TCCCGGTCCCTTGCCTCTGGA -TAMRA-3′), 0.5 μL of ROX Reference Dye II, 9.5 μL of sterile distilled water and 1 μL extracted RNA. The reaction was run in an ABI 7500 machine following the manufacturer’s instructions.

### Histopathology

Lung samples from pigs were collected from the right cranial lobe and fixed in 10% neutral buffered formalin, and 5 μm sections were cut and stained with hematoxylin and eosin as described previously^54^.

### Statistical analysis

Statistical analysis was performed using GraphPad Prism version 5 (GraphPad Software, San Diego, CA, USA). Statistical analyses were performed by one-way analysis of variance, followed by Tukey’s *t*-test and student’s *t*-test. *P* < 0.05 represents a statistically significant difference. All data are expressed as the mean ± standard error of mean (SEM).

### Ethics approval

The study and protocol was approved by the Science and Technology Agency of Jiangsu Province. The approval ID is NKYVET 2015-0066, granted by the Jiangsu Academy of Agricultural Sciences Experimental Animal ethics committee. All efforts were made to minimize animal’s suffering. The immunization, challenge, collection of serum samples and separation of pig PBMCs were performed in strict accordance with the guidelines of Jiangsu Province Animal Regulations (Government Decree No. 45).

## Additional Information

**How to cite this article:** Du, L. *et al*. Assessment of the efficacy of two novel DNA vaccine formulations against highly pathogenic Porcine Reproductive and Respiratory Syndrome Virus. *Sci. Rep.* **7**, 41886; doi: 10.1038/srep41886 (2017).

**Publisher's note:** Springer Nature remains neutral with regard to jurisdictional claims in published maps and institutional affiliations.

## Figures and Tables

**Table 1:** The scores of clinical signs of the pigs after challenge^a^.

Groups	Clinical signs scores (±S.E.M.)^b^
PBS	6.8 ± 0.26A
pcDNA3.1-SynORF5	5.6 ± 0.24B
pcDNA3.1-PoIFN-λ1-SynORF5	4.3 ± 0.26C
BPEI/PLGA-SynORF5	3.9 ± 0.11C
JXA1-R	3.3 ± 0.28D

^a^Within each column, values followed by different letters (A, B, C, and D) are significantly different (*P* < 0.05).

^b^Scores for clinical signs were determined by sum of daily observations of behavior, respiration and cough according to the severity or the illness.
